# Supplementary material for: Rapid evaluation of bioactive Ti-based surfaces using an in vitro titration method
Source: Nat Commun. 2019 May 2;10:2062. doi: 10.1038/s41467-019-09673-1 (PMC6497645; doi:10.1038/s41467-019-09673-1)
Supplement: Supplementary file 1 — Supplementary Information [file 41467_2019_9673_MOESM1_ESM.pdf]

Supplementary Information

Rapid evaluation of bioactive Ti-based surfaces using an *in vitro* titration method

Zhao et al.

Supplementary Methods

Protocols of using calcium titration for the evaluation of bioactivity

Solution preparation

Preparation of titration solutions

Reagent	Purity	MW	Ca stock			P stock		
			mM		Unit	mM		Unit
NaCl	99.5%	58.44	100.0	5.8734	g/L	100.0	5.8734	g/L
$Na_2HPO_4 \cdot 2H_2O$	98.0%	177.99				4.0	0.7265	g/L
$CaCl_2$	97.0%	110.99	20.0	2.2884	g/L			
Tris	100.0%	121.14	20.0	2.4228	g/L	20.0	2.4228	g/L
HCl (1.0 M)	100.0%		17.5	0.1750	L/L	17.5	0.1750	L/L

1. All the solutions are prepared using de-mineralized water.
2. NaCl and Tris-HCl are added to both stock solutions to keep a relatively stable pH and ionic strength background during titration.
3. In the preparation of Ca stock solution, Tris is recommended to be added in the last to prevent high pH environment for possible $Ca(OH)_2$ precipitation (Maximum pH around 10.5 when only Tris is added without HCl).
4. Before experiments, solutions are recommended to be degassed with flowing nitrogen to remove CO_2.

Titration system setup

1. pH meter and Ca probe should be cleaned each time before and after titration experiments for possible precipitates on surface by immersing in dilute acidic solution (e.g. 0.01M HCl).
2. pH electrode combined with temperature probe (InLab Expert Pro, Mettler Toledo) should be calibrated before experiment using commercial standard buffer solutions (3-point calibration).
3. Calcium electrode (perfectION comb Ca combination electrode, Mettler Toledo) should be calibrated using standard 10 mg/L, 100 mg/L, and 1000 mg/L at similar ionic strength of the reaction solution (for the solution design listed in table above, 0.125 M NaCl is added into calcium standard prepared with $CaCl_2$).
4. The polymeric membrane of the calcium-sensitive electrode is recommended to be conditioned before experiments to avoid possible small amount of Ca^{2+} release during the experiments from the membrane itself.

Titration experiment

1. Set thermostatic bath to the desired testing temperature.
2. Place 50 mL P solution into a 100 mL beaker with magnetic stirrer to ensure homogeneity of solution under constant mixing condition.

3. Place pH and Ca electrodes into solution and start data collection. Due to typical initial drifts of both pH and Ca value, titration experiment is recommended to start after 15 minutes from the starting point of data collection.
4. Titrate Ca solution dropwise into P solution at a rate of choice (nominally around 0.1 mL/min). In the current experiment, the flow rate was determined to be 0.077 mL/min.
5. The titration experiment data is collected online with a Mettler Toledo SevenExcellence system. Both free calcium ion concentration and pH value are collected. In the case where calcium electrode is not available, data of pH value is sufficient to determine the calcium phosphate nucleation onset point. However, the adsorption/release of free calcium ions by the testing material cannot be determined.
6. During titration, temperature is controlled within 25.0 ± 1.0 °C.
7. At the end of the experiments, measure again standard Ca solution at 100 mg/L and standard pH solution at 7.01. Even though the calcium electrode is calibrated at the similar ionic strength as the titration solution, a drift in electrode response can often be observed. The free calcium concentration can be adjusted using a linear correction.

Details of the surgical procedure and implant design

Anesthesia

Prior to surgery the sheep were sedated with Buprenorphine (0,01 mg/kg KGW, i.m., Temgesic, Essex Chemie AG, Switzerland) and Xylazin (0,1 mg/kg KGW, i.m., Streuli Pharma AG, Switzerland). General anesthesia was induced with ketamine (3 mg/kg BW, i.v., Ketanarkon 100 ad. us. vet., Streuli Pharma AG, Switzerland), midazolam (0,1 mg/kg KGW, i.v., Midazolam Sintetica, 0,1 mg/kg KGW, i.v., Sintetica S.A., Switzerland) und Propofol (0,4 mg/kg BW, i.v., Propofol 1% MCT Fresenius, Fresenius Kabi AG, Switzerland). A tube was placed into the trachea and the anesthesia was maintained with inhalation of 1–1.5% isoflurane (Attane, Isoflurane ad us. vet., Piramal Enterprises Limited, India) and intravenous application of propofol (0,1 mg/kg BW/h) and ketamine (3mg/kg BW/h) under constant application of fluids (5 ml/kg BW/h, i.v., Ringer-Acetat „Bichsel“, Laboratorium Dr. G. Bichsel AG, Switzerland). Additional monitoring was provided through an ECG, pulsoxymetry, capnography and invasive blood pressure measurement. Analgesia was achieved through an additional epidural anesthesia with morphine (0,1 mg/10 kg BW, Morphin-HCL, Sintetica S.A., Switzerland) at the foramen lumbosacrale during surgery and the injection of carprofen (4 mg/kg BW, i.v., RIMADYL, 1x tgl, Zoetis, Switzerland) for 5 days. Buprenorphine (0,01 mg/kg BW, i.m., Temgesic, Essex Chemie AG, Switzerland) was given perioperatively and continued every 4–6 hours three times after surgery. Antibiotics (penicillin, 30000 IE/kg BW, i.v., 2x dly., Penicillin Natrium Streuli, Streuli Pharma AG, Switzerland and gentamycin, 4 mg/kg BW, i.v., 1x dly., Vetagent, MSD Animal Health GmbH, Switzerland) and Tetanus serum (3000 IE/kg BW, s.c., Intervet, MSD animal health GmbH, Switzerland) were given for prophylaxis.

Surgical operation and postoperative care

The anesthetized sheep were placed in lateral recumbency. The operation area was clipped, washed and sterilized. An incision was made from 3 cm cranial to 10 cm caudal of the midline of the iliac crest. The fascia as well as fat tissue were cut. The medial gluteal muscle and the tensor fasciae latae muscle were bluntly dissected to their origin at the iliac crest. Both muscles together with the deep gluteal muscle were separated from the iliac wing and held back with Langenbeck retractors during the implantation process. A flexible template was placed on the iliac crest to maintain equal implant locations in each

sheep. Six holes were pre-drilled with a depth of 4 mm and a diameter of 6 mm. Then a drill with a drillstop and a 180° tip was used to drill to 7 mm depth. All implant drilled holes displayed a diameter of 6 mm with a flat bottom, ensuring an ideal fit without press-fit effect or peri-implant bone condensation. After a manual implant placement, the protruding 3 mm of each implant were covered with PEEK-caps, avoiding callus growth over the implant tip. Muscles and soft tissues were repositioned and fixed with resorbable sutures (Vicryl 2-0 (3 metric), Johnson & Johnson, Belgium). The skin was closed with medical staplers, before a gauze was placed over the wound. The animal was turned on the other side to repeat the procedure in an identical manner. Postoperatively sheep were held in small boxes for 1.5 weeks, before the skin-staplers were removed and sheep being transferred to larger stalls for the remaining duration of the study. After 2 and 8 weeks the sheep were sacrificed and the samples harvested.

Harvesting of the specimens

Both iliac bones were harvested directly after slaughtering, muscles and surrounding tissue were scraped off and implants and bone were macroscopically assessed for signs of inflammation or loosening. Two radiographs were taken of each iliac bone (55 kV/1s) using an Faxitron X-ray machine (Faxitron X-Ray System, Hewlett&Packard, Kodak X-OMAT MA Film, France). Afterwards, all implants, together with the surrounding bone, were cut into cubical pieces (2 x 2 cm) using a handsaw (Stryker Instruments, USA). Implant blocks for torque testing were wrapped in wet gauze and tested within 6 hours.

Experimental procedures of cell culture tests using osteoblast

Materials preparation

For cell culture experiments, commercial pure (CP) grade 1 titanium discs (Hempel Special Metals, Switzerland) of ⌀10 mm × 2 mm were all machine polished first before any chemical treatment. The polishing was applied on both sides of the discs using silicon carbide P320, P1200, and 1200/4000 abrasive paper consecutively, followed by washing with acetone, water and ethanol in an ultrasonic bath. The NaOH treatment was conducted using 5 M NaOH solution for 24 h at 60°C. Afterwards, the discs were washed using flowing de-ionized water for about 30 s and then dried. The heat treatment was applied at 600°C (heating rate 100°C/h, natural cooling) for an hour in ambient atmosphere. An extra surface is prepared by immersing NaOH-treated Ti disc in standard SBF for 5 days to obtain a surface coating of HA.

Cell viability

Five titanium surfaces (polished Ti, Ti HT, Ti NaOH, Ti NaOH HT, Ti NaOH covered with HA) were tested in cell culture using osteoblast lineage cells from *C57BL/6J* mice, obtained from Charles River Laboratories in Sulzfeld, Germany. The cells were obtained from mouse calvariae using a sequential collagenase digestion method. These cells were cultured in α-minimum essential medium (αMEM, GIBCO BRL Life Technologies, Basel, CH) supplemented with 1% Penicillin/Streptomycin (GIBCO) and 10% FBS (not heat inactivated, Sigma) with or without bone morphogenetic protein 2 (BMP-2), and grown in a humidified 95/5% air/CO_2 atmosphere incubator at 37 °C. After culture on treated Ti surfaces on 48-well plates at a density of 2000/well for 3, 7 and 14 days, cell proliferation was determined using a standard 2,3-*bis* [2-methoxy-4-nitro-5-sulfophenyl]-2*H*-tetrazolium-5-carboxanilide inner salt (XTT) assay kit (Roche Applied Science). The measurement is based on the

cleavage of the yellow XTT salt to the formation of an orange formazan dye by metabolically active cells. XTT labeling reagent was added to each well after cell culture. After incubation for 4 h in an incubator at 37°C and 5% CO_2, 150 µL of the medium was transferred to a 96-well plate for measurement. The amount of produced formazan was quantified by measuring absorbance at 470 nm.

Alkaline phosphatase (ALP) activity

Osteoblasts from *C57BL/6J* mice (2 × 10^3 per well) were grown in 48-well tissue culture plates on different titanium discs at 37 °C in an incubator with 5% CO_2. At the end of the culture, the cells were washed with PBS and lysed in 20 µL 0.1% Triton-X100 in water in three freeze/thaw cycles. After incubation with 0.1 ml of 3 mM *p*-nitrophenylphosphate (pNPP, Sigma) in 1 M ethanolamine, pH 9.5, for 30 min at room temperature, the reaction was stopped with EDTA, and the absorption was measured at 405 nm with a multi-well spectrophotometer. The enzyme activity was normalized against the cell number measured by XTT assay and is given as A_{405}/A_{470}.

Supplementary Figures

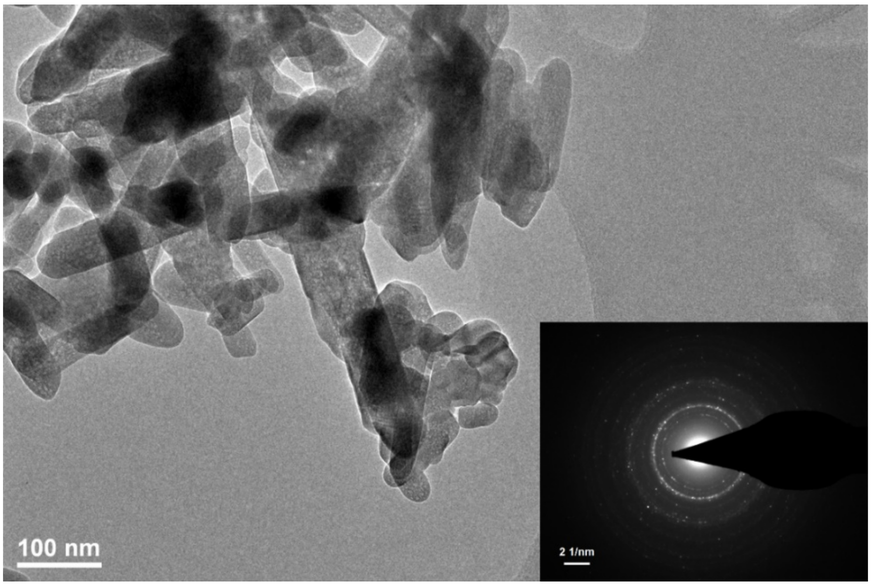

Supplementary Figure 1. Morphology of a commercial HA powder observed in TEM. Inset shows the selected area electron diffraction (SAED) pattern of the same powder showing multiple crystalline domains.

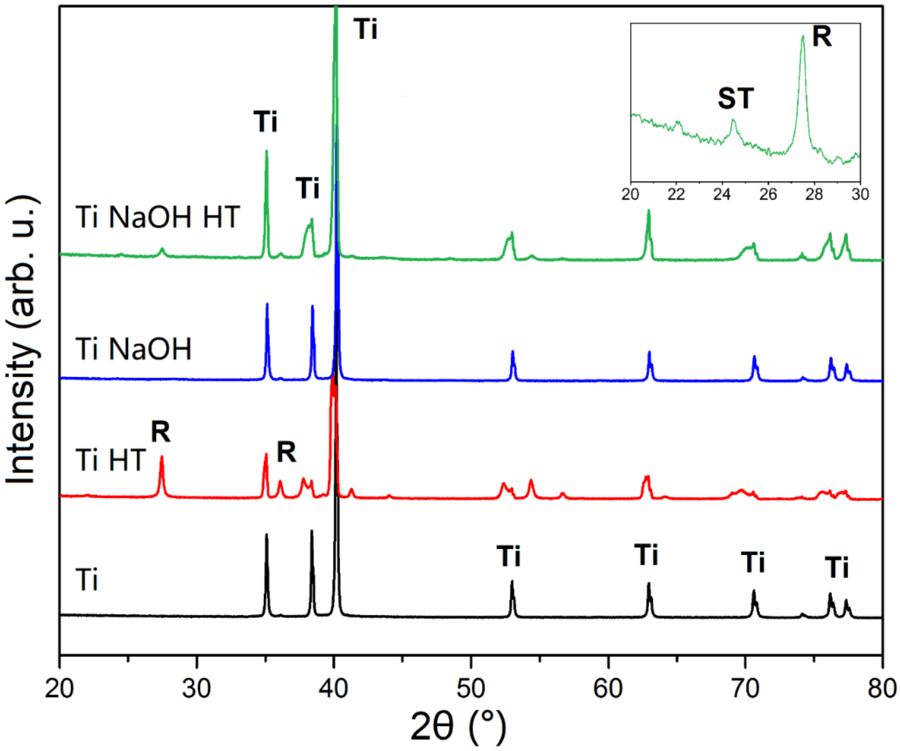

Supplementary Figure 2. XRD patterns of four Ti powders after chemical treatment. Pure titanium (Ti) is identified for commercial titanium powder. After heat treatment, TiO_2 rutile (01-089-4920), denoted as R, is found on the surface in addition to Ti. No significant change of XRD pattern is observed after NaOH treatment, mostly due to poor crystallinity of the porous layer, thus masked by the diffraction pattern from the bulk of the powder. Further heat treatment results in a mixture of TiO_2 rutile (01-089-4920), sodium titanate ST: $Na_2Ti_6O_{13}$ (00-037-0951) and Ti. The sodium titanate peak can be better visualized by the inset showing a zoomed-in focused XRD scan from 20–30°. It is possible that Ti_6O (01-072-1807) is also formed after heat treatments due to the split of the Ti peak at 37°.

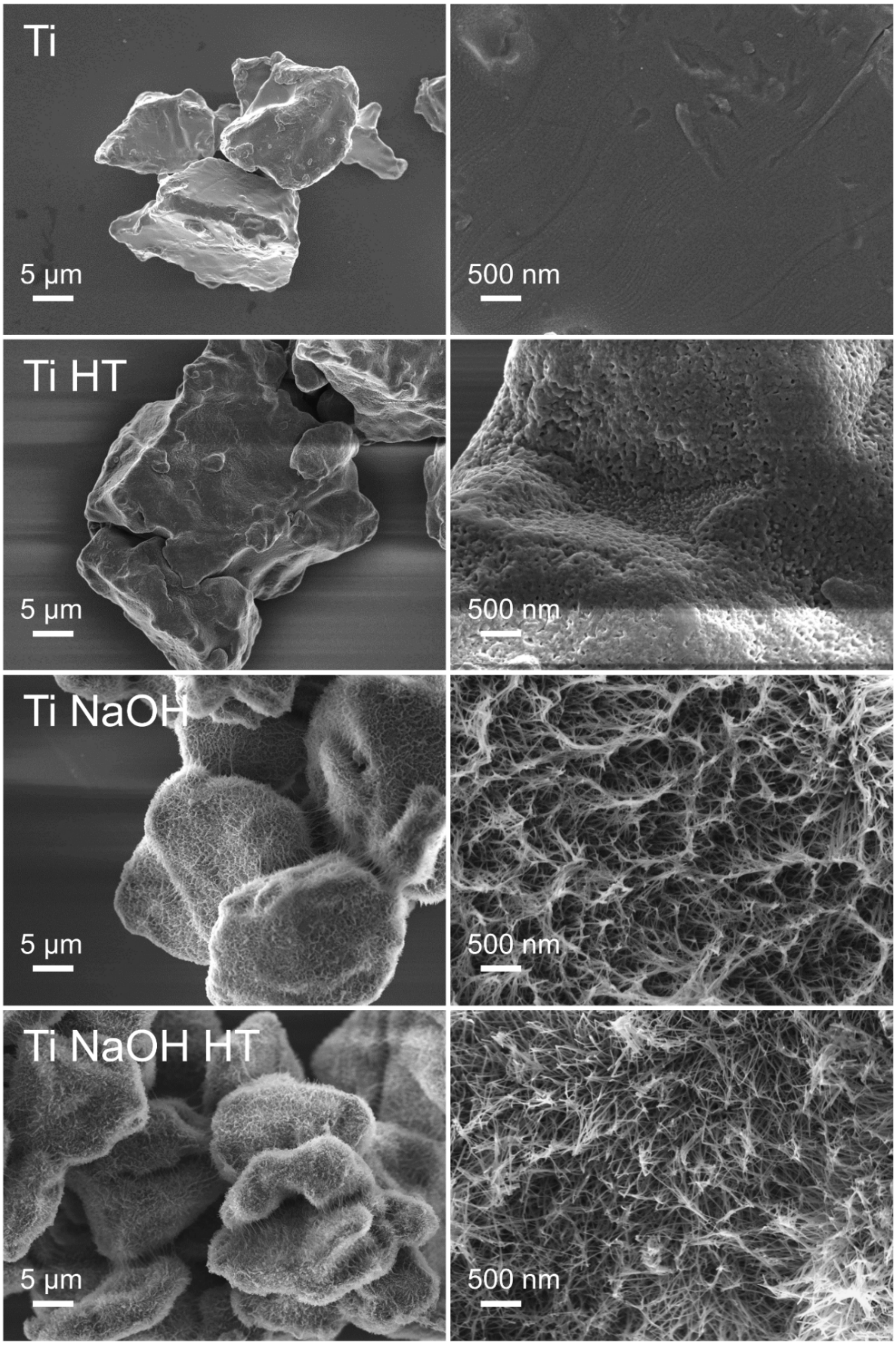

Supplementary Figure 3. SEM images of titanium powders after chemical treatment. The right column is a higher magnification image of the same surface on the left column.

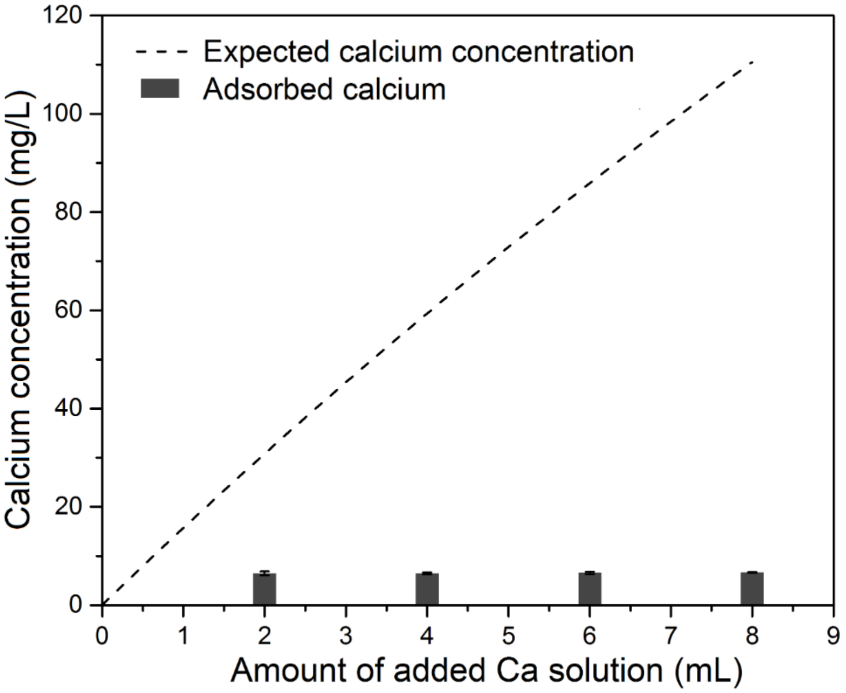

Supplementary Figure 4. Column chart of adsorbed amount of Ca at four different Ca concentrations. The expected total calcium concentration is given by the dash line. The error bars are generated with sample size of *n* = 2 with distinct samples.

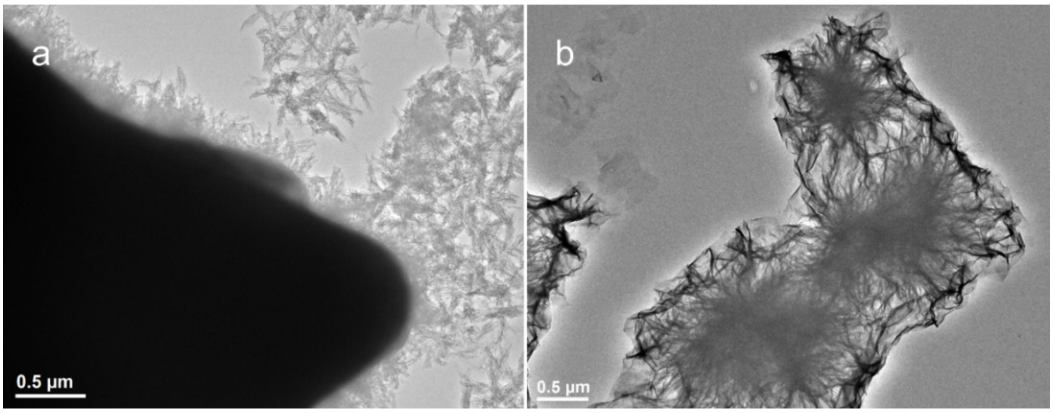

Supplementary Figure 5. Titration product using Ti-based powder. (a) TEM image of nucleation product of Ti powder near 230 min corresponding to the second peak in free calcium profile showing both OCP crystals attached to the Ti particle and unattached OCP particles. (b) TEM of nucleation product in the solution at the end of titration between 250 – 300 min showing aggregated nano-flakes resembling typical HA morphology in the solution, unattached to any Ti particles.

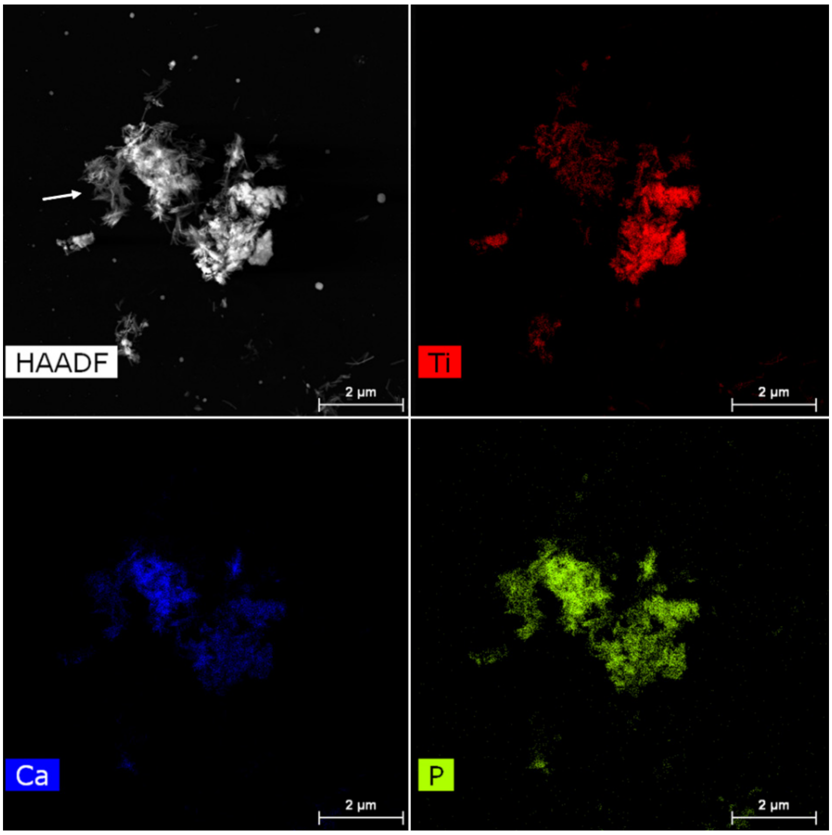

Supplementary Figure 6. Elemental distribution of Ti NaOH titration products. Images include high-angle annular dark-field (HAADF) image and EDX elemental mapping of nucleation product 5–10 min before reaching the free calcium peak in the titration experiment using Ti NaOH. Crystalline CaP species (indicated by a white arrow) resembling OCP can already be seen, together with pieces of Ti NaOH, as revealed by elemental distribution.

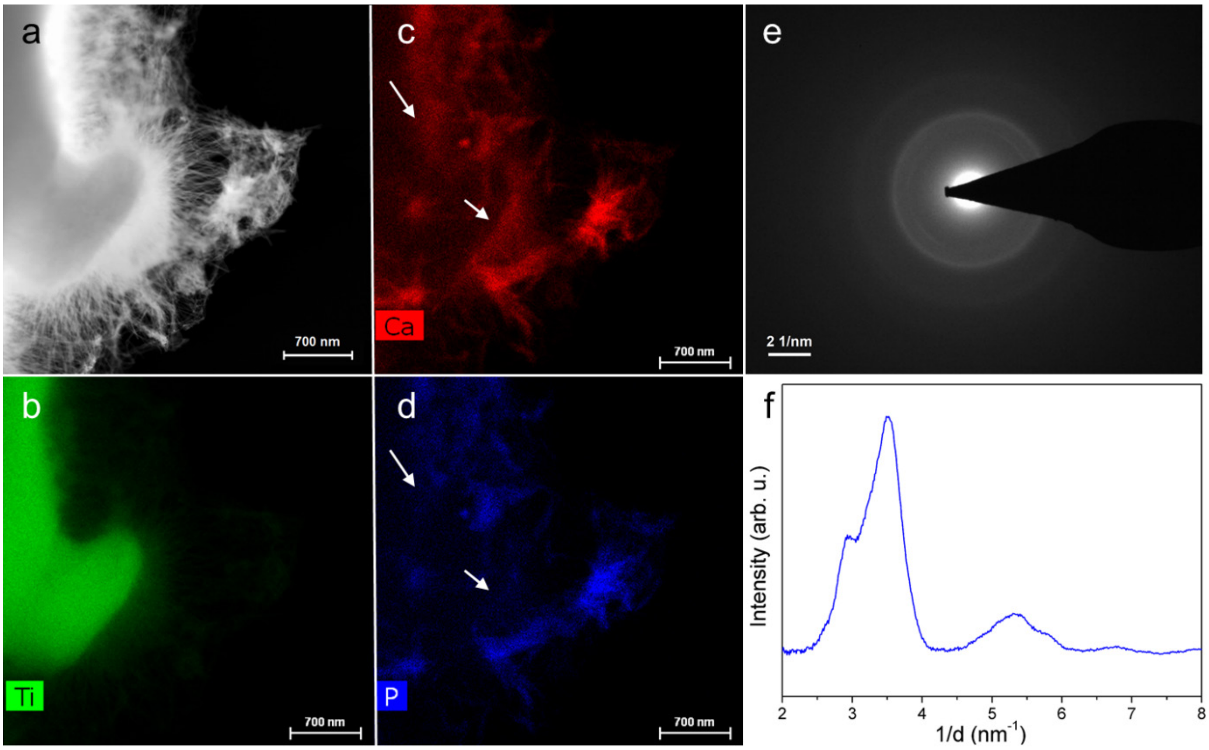

Supplementary Figure 7. HAADF image (a) and corresponding EDX mapping (b), (c) and (d) of nucleation product at the peak in the titration experiment with Ti NaOH powder. The arrows indicate regions where Ca is detected but not P, showing Ca incorporation into the porous surface layer of Ti NaOH powder likely due to ionic exchange. Crystalline calcium phosphate species are found to be embedded in the porous structure, as indicated by the electron diffraction pattern (e) and corresponding radial density distribution (f).

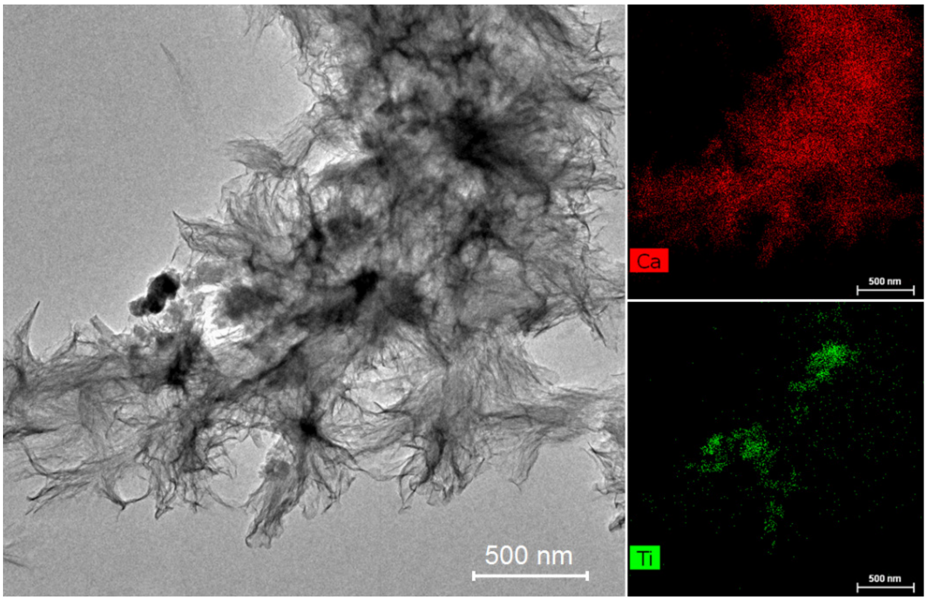

Supplementary Figure 8. TEM image and corresponding EDX mapping (in the STEM mode) of nucleation product after the peak in free Ca profile. The morphology resembles HA morphology and particles containing Ti is found at the core of the structure, possibly playing the role of initiating the nucleation.

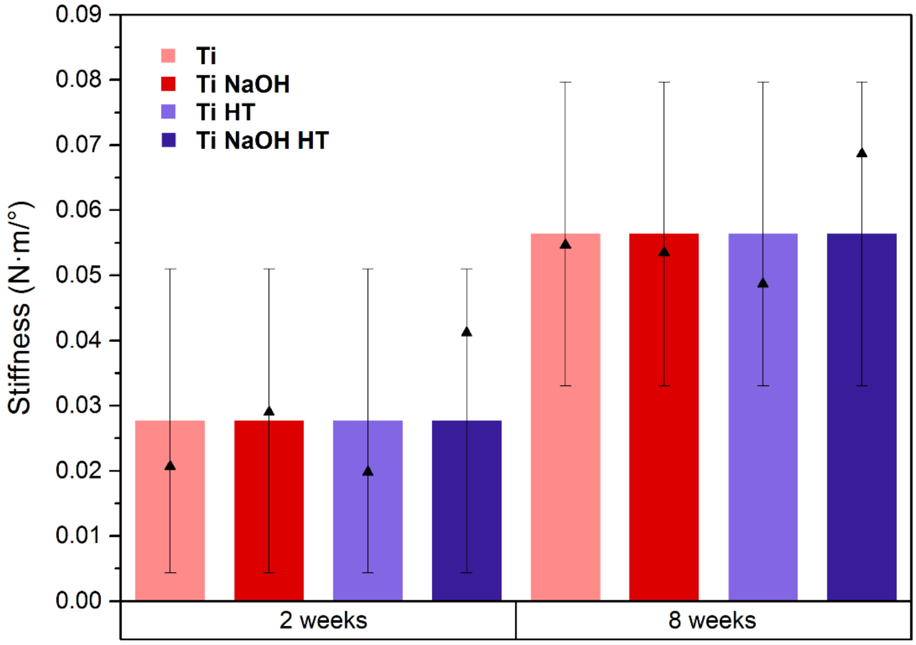

Supplementary Figure 9. Control chart for the stiffness. The columns give the fitted values according to a multifactorial statistical analysis with error bars corresponding to the 95% confidence interval. The solid triangles give the experimental mean values based on 6 replicates. Factor time delay ($p < 0.01$) is identified to be statistically significant. Interaction between time delay and position (L/R) is also identified to be significant ($p < 0.01$), however globally the factor position itself is not significant. This is likely due to inter-animal variability as different animals are used for different time delays and for only one implantation position (each combination of time and position corresponds to 2 different sheep). Thus this factor is treated as noise in the analysis and thus not included in the control chart.

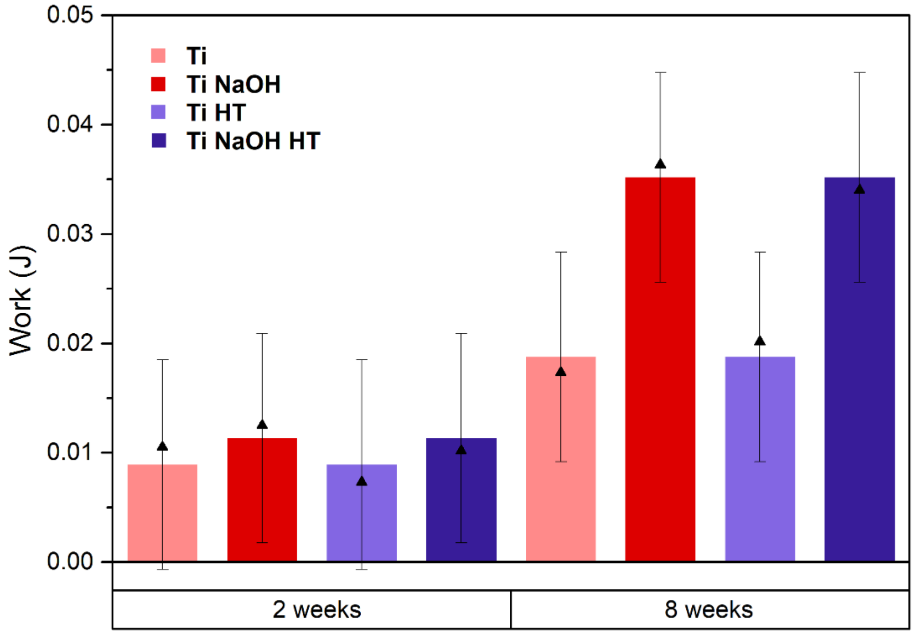

Supplementary Figure 10. Control chart for the energy to yield point. The columns give the fitted value according to a multifactorial statistical analysis with error bars corresponding to the 95% confidence interval. The solid triangles give the experimental mean values based on 6 replicates. Factor NaOH treatment ($p < 0.01$), time delay ($p < 0.01$) and the interaction of the two factors ($p < 0.05$) are identified to be statistically significant.

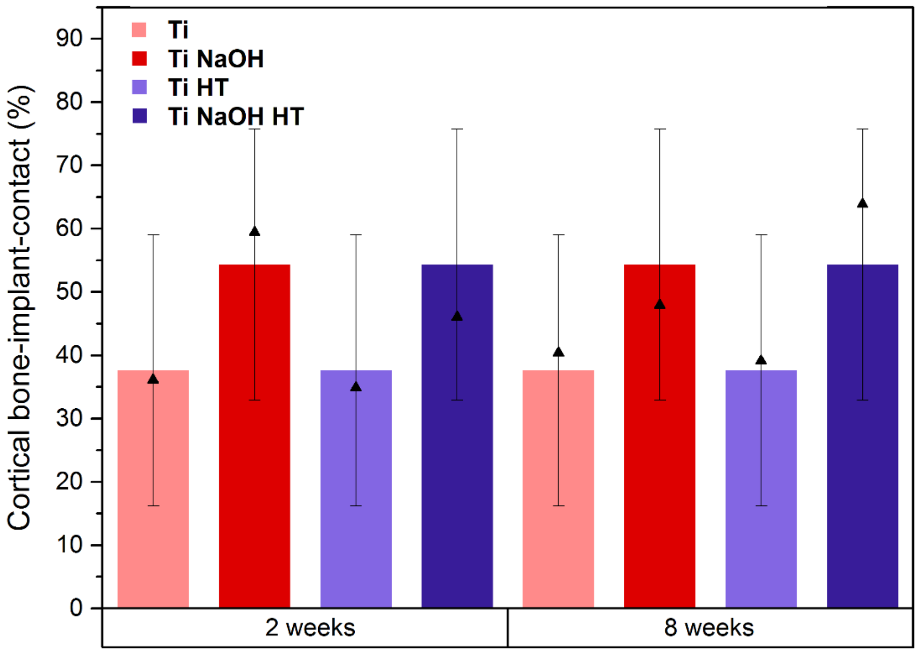

Supplementary Figure 11. Control chart for the cortical BIC. The columns give the fitted value according to a multifactorial statistical analysis with error bars corresponding to the 95% confidence interval. The solid triangles give the experimental mean values based on 6 replicates. Only factor NaOH treatment ($p < 0.01$) was identified to positively affect the BIC in the cortical bone.

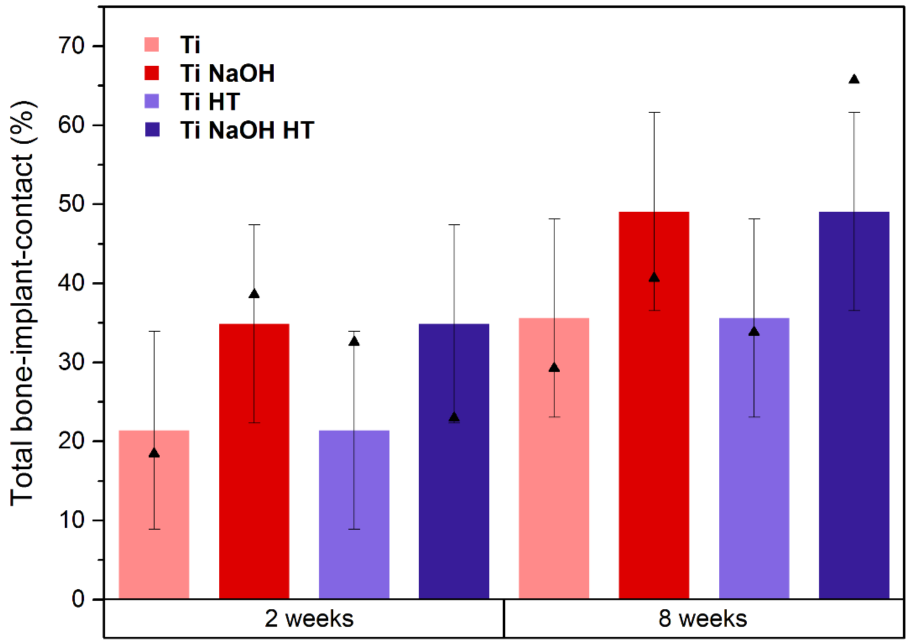

Supplementary Figure 12. Control chart for the total BIC. The columns give the fitted value according to a multifactorial statistical analysis with error bars corresponding to the 95% confidence interval. The solid triangles give the experimental mean values based on 6 replicates. Factor NaOH treatment ($p < 0.01$), time delay ($p < 0.01$) and the interaction of the two factors ($p < 0.05$) are identified to be statistically significant. Other higher order interactions are treated as noise due to insignificant effect of heat treatment, position and replicate.

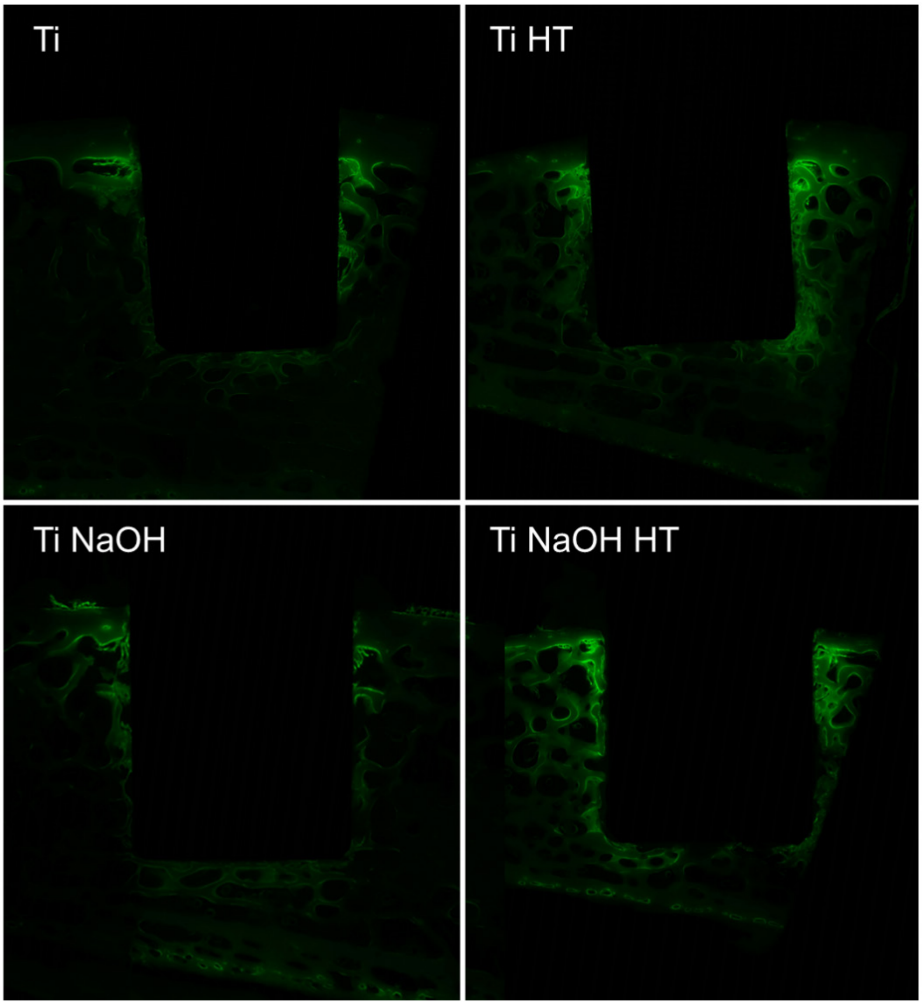

Supplementary Figure 13. Fluorochrome labeling of titanium implants 48–72 h before sacrifice for the 2-week group. The visible green regions represent the bone deposition roughly 2 weeks after implantation. Since total 6 replicates are used for each implant type, the images chosen are not necessarily a quantitatively average representative of the bone remodeling process for each implant type. However, the same sheep (#06) is chosen in this case (choice based on image quality) to lower inter-animal variability for enhanced consistency.

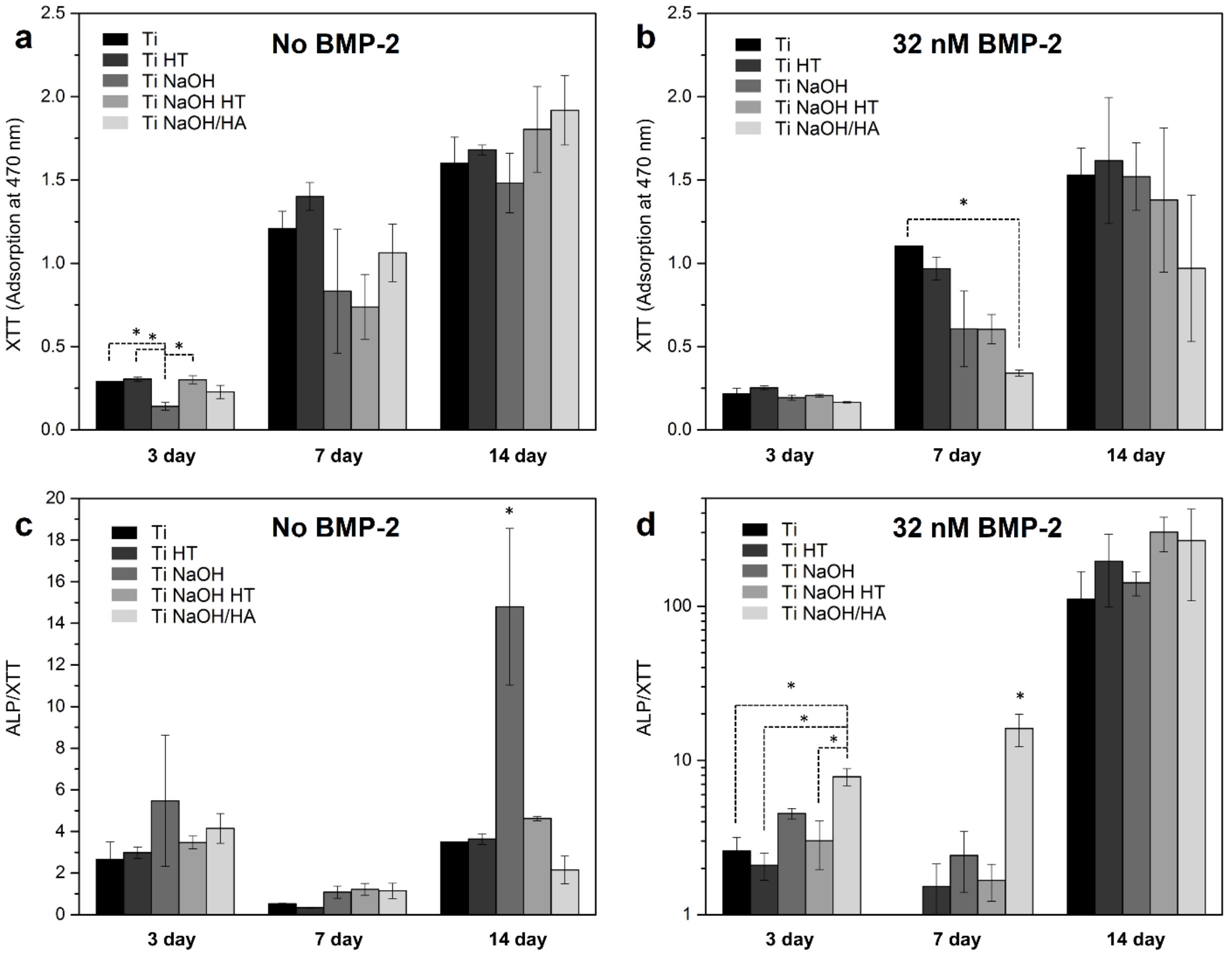

Supplementary Figure 14. Cell viability and ALP activity. Cell viability tested with XTT at different time points without BMP-2 (a) and with BMP-2 (b). ALP activity normalized with XTT without BMP-2 (c) and with BMP-2 (d). Statistically significant difference between groups but within each time point is indicated by a star (*: $p < 0.01$) following the Tukey's multiple comparisons ($n = 2$ with distinct samples). Notice that figure d adopts a log-scale y axis to better visualize the data.

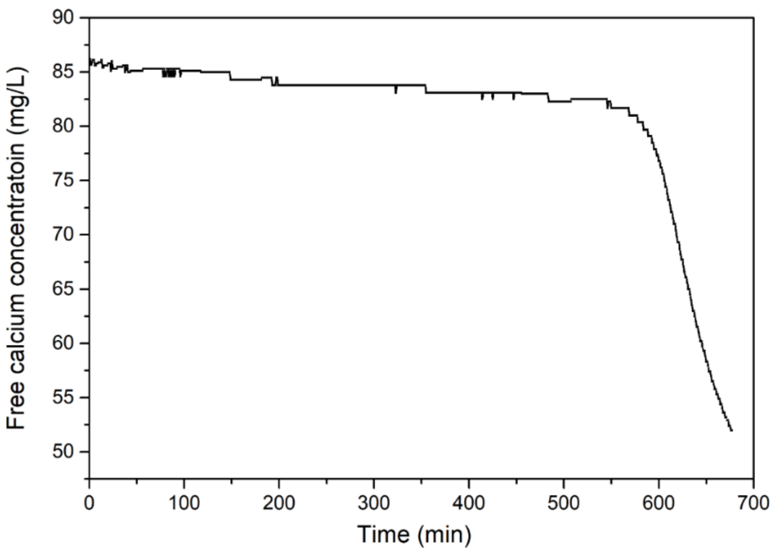

Supplementary Figure 15. The evolution of free calcium profile. Raw data showing a slow decrease of free calcium concentration as a function of time and the eventual drastic precipitation after 10 h. The solution used is prepared by mixing 50 mL P stock solution with 5 mL Ca stock solution. Magnetic stirring is applied during the test.

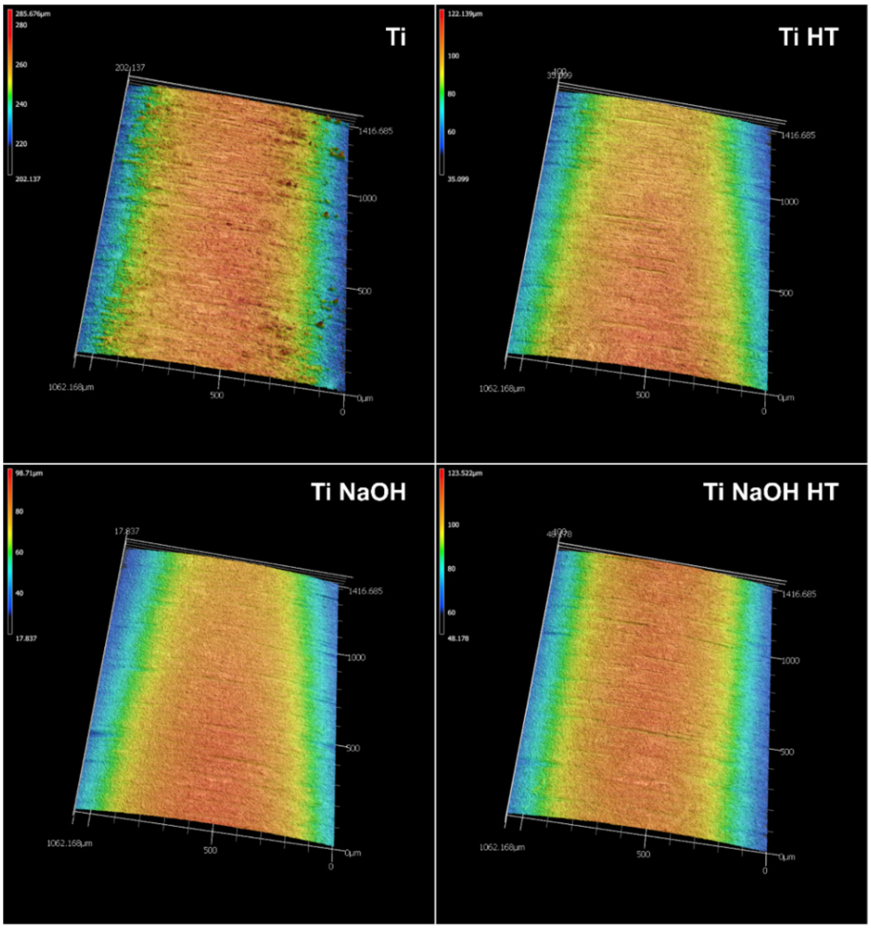

Supplementary Figure 16. 3D surface profiles of four Ti bars. The observation area is ~1.0 mm x 1.5 mm on the side of the Ti bars (6 mm in diameter), which revealed the curvature of the side surface. The profiles were obtained with 3D Laser Scanning Microscope, VK-X200, Keyence, USA.

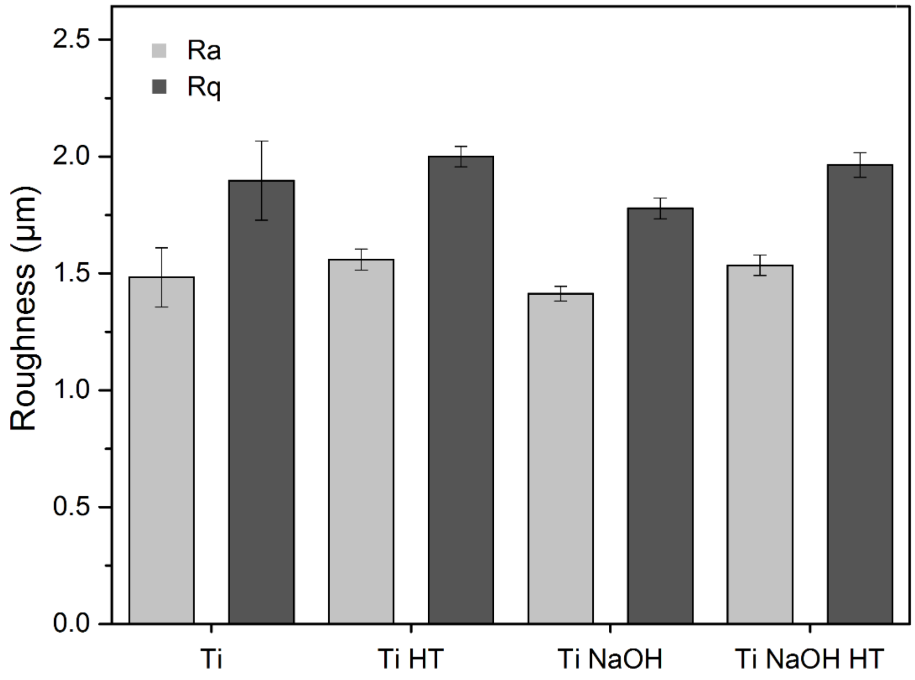

Supplementary Figure 17. Absolute surface roughness R_a and root-mean-square roughness R_q of all four surfaces. The average and error bars are generated based on 8 distinct sample lines of ~1.5 mm in length that are perpendicular to the polishing features from the images shown in Supplementary Fig. 16. A cutoff wavelength of 0.08 mm was used for the correction of waviness for all analyses.

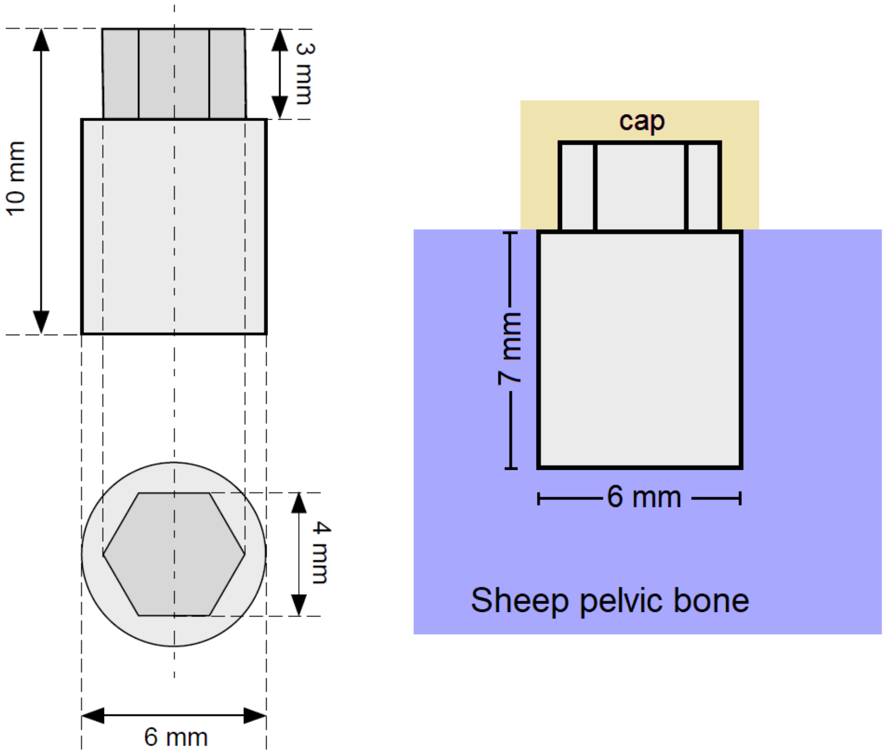

Supplementary Figure 18. Design of implants used for the torque tests.

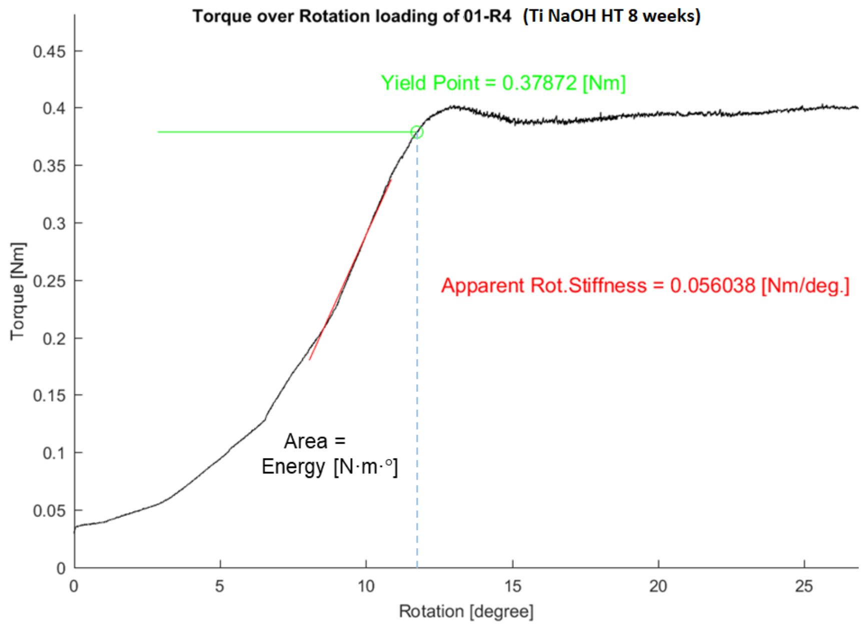

Supplementary Figure 19. An example of data analysis of the torque tests. The example is taken from a Ti NaOH HT surface implanted for 8 weeks. The rotational stiffness is extracted as the slope of the linear region in the torque curve. The yield point is defined as the torque value at which the slope of the curve falls to 50% of that in the linear region. The area under the curve (energy) till the yield point then gives the work needed to reach implant failure.

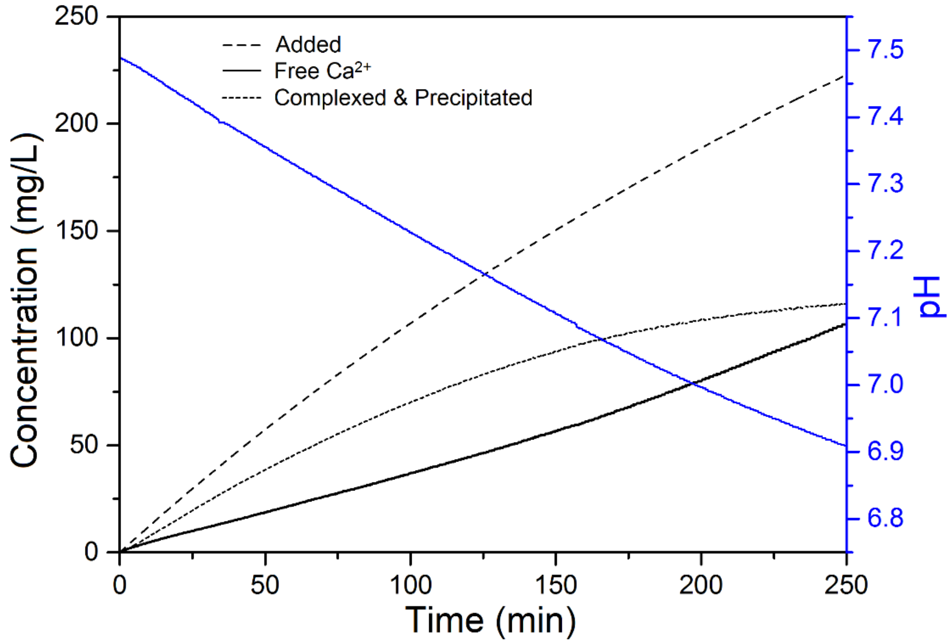

Supplementary Figure 20. Titration experiment using 0.1 g commercial nanocrystal HA powders. No major nucleation event is observed. Complexed & precipitated Ca concentration continue to increase indicating a continuous growth of HA on HA seeds from the beginning of the titration. The same setup and parameters as described in the method section were used.

Supplementary Tables

Supplementary Table 1. Measurement of free calcium changes due to the addition of Ti NaOH powder.

Ca stock vol. (mL)	Total Ca		Ca adsorption (mg/L)		Avg.		Std.	
	mM	mg/L	Mes. 1	Mes. 2	mg/L	mg	mg/L	mg
2	0.77	30.8	6.15	6.75	6.45	0.335	0.42	0.032
4	1.48	59.4	6.28	6.56	6.42	0.347	0.19	0.020
6	2.14	85.9	6.38	6.71	6.54	0.366	0.23	0.030
8	2.76	110.6	6.60	6.70	6.65	0.386	0.07	0.011

Supplementary Table 2. Implant distribution for the 8-week group. Implants for torque tests are shaded in red and implants for BIC are shaded in grey. Implants are labeled as: 1: Ti, 2: Ti HT, 3: Ti NaOH, 4: Ti NaOH HT.

**Time**<br>8 weeks	**Position**<br>*Left pelvis*						*Right pelvis*					
**Sheep**	L1	L2	L3	L4	L5	L6	R1	R2	R3	R4	R5	R6
#01	1	2	3	4	1	2	1	2	3	4	1	2
#02	3	4	1	2	3	4	3	4	1	2	3	4
#03	2	3	4	1	2	1	2	3	4	1	2	1
#04	4	1	2	3	4	3	4	1	2	3	4	3

Supplementary Table 3. Implant distribution for the 2-week- group. Implants for torque tests are shaded in red and implants for BIC are shaded in grey. Implants are labeled as: 1: Ti, 2: Ti HT, 3: Ti NaOH, 4: Ti NaOH HT.

**Time**<br>2 weeks	**Position**<br>*Left pelvis*						*Right pelvis*					
**Sheep**	L1	L2	L3	L4	L5	L6	R1	R2	R3	R4	R5	R6
#05	1	2	3	4	1	2	1	2	3	4	1	2
#06	3	4	1	2	3	4	3	4	1	2	3	4
#07	2	3	4	1	2	1	2	3	4	1	2	1
#08	4	1	2	3	4	3	4	1	2	3	4	3

Supplementary Table 4. Design of cell culture experiments using osteoblast

Factor	Definition	Level 1	Level 2	Level 3	Level 4	Level 5
A	Sample	Ti	Ti HT	Ti NaOH	Ti NaOH HT	Ti NaOH (HA)
B	BMP-2	No	Yes			
C	Time delay (d)	3	7	14		
D	Replicate	1	2			

Supplementary Table 5. Raw data from the torque tests for all 48 implants

#	NaOH	HT	Time	Position	Replicate	Stiffness [Nm/°]	Yield [Nm]	Energy [J]
1	No	No	2w	L	1	0.0064	0.0584	0.0009
2	Yes	No	2w	L	1	0.0148	0.1608	0.0151
3	No	Yes	2w	L	1	0.0231	0.1089	0.0042
4	Yes	Yes	2w	L	1	0.0026	0.0941	0.0202
5	No	No	8w	L	1	0.0838	0.3265	0.0143
6	Yes	No	8w	L	1	0.0725	0.3582	0.0219
7	No	Yes	8w	L	1	0.0250	0.2371	0.0228
8	Yes	Yes	8w	L	1	0.0491	0.4293	0.0413
9	No	No	2w	R	1	0.0281	0.2281	0.0198
10	Yes	No	2w	R	1	0.0207	0.1352	0.0076
11	No	Yes	2w	R	1	0.0160	0.1485	0.0105
12	Yes	Yes	2w	R	1	0.0435	0.1984	0.0108
13	No	No	8w	R	1	0.0388	0.4025	0.0450
14	Yes	No	8w	R	1	0.0456	0.4748	0.0532
15	No	Yes	8w	R	1	0.0345	0.2189	0.0150
16	Yes	Yes	8w	R	1	0.0560	0.3787	0.0282
17	No	No	2w	L	2	0.0356	0.0367	0.0002
18	Yes	No	2w	L	2	0.0126	0.1983	0.0265
19	No	Yes	2w	L	2	0.0094	0.0909	0.0060
20	Yes	Yes	2w	L	2	0.0767	0.0205	0.0000
21	No	No	8w	L	2	0.0661	0.1812	0.0048
22	Yes	No	8w	L	2	0.0637	0.4676	0.0437
23	No	Yes	8w	L	2	0.0712	0.3369	0.0169
24	Yes	Yes	8w	L	2	0.1181	0.5262	0.0250
25	No	No	2w	R	2	0.0109	0.1421	0.0162
26	Yes	No	2w	R	2	0.1031	0.2731	0.0092
27	No	Yes	2w	R	2	0.0135	0.1416	0.0126
28	Yes	Yes	2w	R	2	0.0949	0.1618	0.0034
29	No	No	8w	R	2	0.0147	0.1390	0.0103
30	Yes	No	8w	R	2	0.0586	0.4959	0.0482
31	No	Yes	8w	R	2	0.0226	0.1693	0.0110
32	Yes	Yes	8w	R	2	0.0537	0.3499	0.0257
33	No	No	2w	L	3	0.0246	0.2012	0.0177
34	Yes	No	2w	L	3	0.0064	0.0835	0.0051
35	No	Yes	2w	L	3	0.0428	0.1500	0.0053
36	Yes	Yes	2w	L	3	0.0173	0.1614	0.0138
37	No	No	8w	L	3	0.1050	0.4141	0.0187
38	Yes	No	8w	L	3	0.0363	0.3269	0.0320
39	No	Yes	8w	L	3	0.1007	0.3690	0.0144
40	Yes	Yes	8w	L	3	0.0926	0.4058	0.0222
41	No	No	2w	R	3	0.0183	0.1294	0.0084
42	Yes	No	2w	R	3	0.0163	0.1584	0.0120
43	No	Yes	2w	R	3	0.0151	0.1009	0.0045
44	Yes	Yes	2w	R	3	0.0112	0.1357	0.0128
45	No	No	8w	R	3	0.0188	0.1526	0.0110
46	Yes	No	8w	R	3	0.0436	0.2795	0.0187
47	No	Yes	8w	R	3	0.0373	0.3823	0.0406
48	Yes	Yes	8w	R	3	0.0421	0.4459	0.0619

Supplementary Table 6. Raw data from the histology analysis for all 48 implants

#	NaOH	HT	Time	Position	Replicate	BIC cortical [%]	BIC cancellous [%]	BIC total [%]
1	No	No	2w	L	1	28.06	16.19	18.11
2	Yes	No	2w	L	1	28.57	71.56	67.18
3	No	Yes	2w	L	1	53.45	27.63	32.25
4	Yes	Yes	2w	L	1	0	13.75	13.75
5	No	No	8w	L	1	24.91	25.7	25.59
6	Yes	No	8w	L	1	51.58	39.68	40.29
7	No	Yes	8w	L	1	39.08	37.69	37.96
8	Yes	Yes	8w	L	1	43.93	66.02	63.85
9	No	No	2w	R	1	0	23.24	15.17
10	Yes	No	2w	R	1	40	30.28	31.1
11	No	Yes	2w	R	1	37.43	17.38	20.22
12	Yes	Yes	2w	R	1	23.45	7.42	8.54
13	No	No	8w	R	1	38.29	30.15	31.86
14	Yes	No	8w	R	1	24.59	24.87	24.83
15	No	Yes	8w	R	1	21.67	37.12	34.97
16	Yes	Yes	8w	R	1	62.16	64.27	63.95
17	No	No	2w	L	2	45	18.07	19.16
18	Yes	No	2w	L	2	51.84	19.22	25.01
19	No	Yes	2w	L	2	0	25.29	23.3
20	Yes	Yes	2w	L	2	74.77	17.46	26.53
21	No	No	8w	L	2	18.18	20.78	20.48
22	Yes	No	8w	L	2	67.37	59.42	60.61
23	No	Yes	8w	L	2	14.18	8.81	9.21
24	Yes	Yes	8w	L	2	64.16	69.59	68.88
25	No	No	2w	R	2	26.6	4.85	7.99
26	Yes	No	2w	R	2	79.71	39.16	44.87
27	No	Yes	2w	R	2	72.13	42.75	48.75
28	Yes	Yes	2w	R	2	36.25	28.77	29.77
29	No	No	8w	R	2	57.84	25.88	29.17
30	Yes	No	8w	R	2	34.09	37.45	36.83
31	No	Yes	8w	R	2	33.87	36.74	36.55
32	Yes	Yes	8w	R	2	76.2	71.13	72.1
33	No	No	2w	L	3	36.54	17.02	20.67
34	Yes	No	2w	L	3	67.63	12.42	19.57
35	No	Yes	2w	L	3	46.23	45.17	45.47
36	Yes	Yes	2w	L	3	89.82	28.08	35.56
37	No	No	8w	L	3	69.7	39.15	45.74
38	Yes	No	8w	L	3	40.3	36.66	37.32
39	No	Yes	8w	L	3	45.65	21.07	26.16
40	Yes	Yes	8w	L	3	58.42	38.89	41.88
41	No	No	2w	R	3	80.43	24.25	29.64
42	Yes	No	2w	R	3	88.78	35.36	43.62
43	No	Yes	2w	R	3	0	25.44	25.44
44	Yes	Yes	2w	R	3	52.17	19.5	23.8
45	No	No	8w	R	3	33.49	21.08	22.55
46	Yes	No	8w	R	3	69.59	38.11	43.91
47	No	Yes	8w	R	3	80.29	52.09	58.2
48	Yes	Yes	8w	R	3	78.28	84.3	83.48

Supplementary Note 1

Thermodynamic model

The thermodynamic model is based on a reported model with updated thermodynamic data [1]. As a summary, calculations are performed based on the material balance equations and the thermodynamic equilibria applied to dissolution and complex formation reactions. The activity coefficients of the ionic species are calculated using the extended Debye-Hückel formula [2],

$$-log\gamma_X = \frac{Az_X^2\sqrt{I}}{1 + Ba_X\sqrt{I}} \quad (1)$$

where Z_X is the charge of the dissolved species X. I is the ionic strength of the solution, defined by

$$I = \frac{1}{2}\sum_X z_X^2[X] \quad (2)$$

[X] is the concentration of species X;
a_X(nm) is the radius of the species X;
A and B are two constants given by the formulas:

$A = 1.8246 \cdot 10^6 \times (\varepsilon T)^{3/2}$ [$L^{1/2} \times K^{3/2} \times mole^{-3/2}$]
$B = 502.9 \times (\varepsilon T)^{1/2}$ [$nm^{-1} \times L^{1/2} \times K^{1/2} \times mole^{-1/2}$]

ε is the dielectric constant of the solvent calculated by the formula:

$\varepsilon = 251.629 - 0.803T + 0.000744T^2$

T [K] is the absolute temperature.

The solver function of Microsoft Excel is used to iteratively solve the activities of the reference ions (namely: Na^+, K^+, Mg^{2+}, Ca^{2+}, HPO_4^{2-}, CO_3^{2-}, SO_4^{2-}, Cl^-…) until selected constraints are met, such as the analytical concentrations of dissolved elements and the equilibrium of selected dissolution reactions etc.
The relative supersaturation σ is calculated using the ratio of the activity product of ion units composing the crystal to the corresponding solubility product K_{sp} considering the number of ion units υ

$$\sigma = S - 1 = \left(\frac{IP}{K_{sp}}\right)^{\frac{1}{\upsilon}} - 1 \quad (3)$$

Supplementary Note 2

Details of statistical model and analysis

The following mathematical equation is used to fit the experimental data, taking into account the significant effects and interactions of the deterministic factors investigated in the study:

$$
\begin{aligned}
y = \mu &+ \beta_A X_A + \beta_B X_B + \beta_{AB} X_A X_B + \beta_C X_C + \beta_{AC} X_A X_C + \beta_{BC} X_B X_C \\
&+ \beta_{ABC} X_A X_B X_C + \beta_D X_D + \beta_{AD} X_A X_D + \beta_{BD} X_B X_D + \beta_{ABD} X_A X_B X_D \\
&+ \beta_{CD} X_C X_D + \beta_{ACD} X_A X_C X_D + \beta_{BCD} X_B X_C X_D + \beta_{ABCD} X_A X_B X_C X_D + \varepsilon
\end{aligned}
$$

where:

- y is the observed response
- μ is the global average of the observations
- X_N = -1, 0, 1 is the coded level of factor **N**
- β_R are the regression coefficients of the model :
  - $R = A, B, C, D$ for the main effects of factors **A**, **B**, **C** and **D**, respectively ;
  - $R = AB, AC, \ldots$ for the first order interactions between factors **A** and **B**, **A** and **C, …** respectively ;
  - $R = ABC, ABD, \ldots$ for the second order interactions between factors **A**, **B** and **C**, **A**, **B** and **D**, … respectively ;
  - $R = ABCD$ for the third order interactions between factors **A**, **B**, **C** and **D**.
- ε is the random experimental error, supposed to be normally distributed around 0, with a standard deviation = σ.

The significance of each regression coefficient according to the accepted Type I two-sided error risk (α) is tested with ANOVA: whenever the error risk p is found to be $< \alpha$, the corresponding regression coefficient is accepted to be significantly $\neq 0$.

Full details about the design and analysis of multifactorial designs can be found in reference [3].

Supplementary Discussion

Adsorption of free calcium by chemically treated Ti powder

The free calcium concentrations of different solutions were measured using a calcium-selective electrode (perfectION Ca Combination Electrode, Mettler Toledo), calibrated with calcium standards at 10 mg/L, 100 mg/L and 1000 mg/L at similar ionic strength. Each adsorption experiment is conducted separately using individual samples. 50 mL stock solution containing NaCl and Tris-HCl at the same concentration as phosphate stock solution described in the main text but without the presence of phosphate is mixed with Ca stock solution of 2, 4, 6, 8 mL, respectively. This is to mimic the titration experiment at different time points. At the current titration rate at 0.077 mL/min, these volumes of Ca solution correspond to 26, 52, 78, 104 min in the titration experiment, respectively.

After a stable reading is achieved, 0.1 g of Ti NaOH powder is added and changes in free calcium ion concentration is monitored. Afterwards the results are normalized to obtain the adsorbed amount at different Ca concentration. Due to the absence of phosphate, essentially 100% of total Ca exists as free Ca^{2+} thus expected Ca^{2+} value can be simply obtained using the total Ca concentration. All adsorption experiments were conducted at the same temperature and pH range as the titration experiment. The absence of phosphate prevents the possible nucleation upon addition of Ti NaOH powders thus the decrease of Ca^{2+} can be fully attributed to the adsorption & uptake of Ca^{2+} ions of Ti NaOH powder with a porous surface. All experiments were conducted with two repetitions. Supplementary Fig. 4 shows the adsorption of free Ca^{2+} at different Ca concentration with data given in Supplementary Table 1. It can be seen that the adsorbed concentration stays relatively constant near 6.50 mg/L at different Ca concentration. However, due to the slight difference in total solution volume since different amount of Ca stock solution is added, the total weight of adsorbed Ca shows a slight increasing trend from 0.335 ± 0.032 mg to 0.386 ± 0.011 mg (two replicates each). Therefore, 6.50 mg/L of adsorption is used in the calculation of degree of supersaturation in the case of Ti NaOH powder. Exact speciation at the nucleation onset point is difficult to be fully modeled considered the complexity of various possible Ca-P species in the system but using the adsorbed value from the current experiments provides a reasonable estimate.

In the case of Ti NaOH HT powder at 0.1 g, only data is collected at 2 mL Ca solution addition corresponding to total 0.77 mM Ca. The adsorbed amount is determined to be 0.50 ± 0.07 mg/L (two replicates), considerably lower than that of Ti NaOH as most exchangeable Na^{+} is immobilized in different sodium titanate phases after heat treatment. In the case of Ti and Ti HT at 0.1 g, no detectable adsorption of free Ca^{2+} is found with an instrument detection limit of 0.1 mg/L. It is worth mentioning that the findings here are also supported by Fig. 3 in the main text. In the case of Ti and Ti HT, both measured free Ca^{2+} and complexed & precipitated Ca rise almost linearly at the beginning of the titration, indicating little Ca adsorption, whereas in the case of Ti NaOH, the slope of measured free Ca^{2+} changes at the beginning and the complexed & precipitated Ca given by the dotted line quickly rise to a certain value, indicating an adsorption of Ca that occurs at the beginning of calcium addition.

Practical considerations for the use of the titration method and limitation

In this work, a simple two-step formation process of CaP crystals was revealed in the titration system. However, the exact solution speciation especially before the first nucleation event can in fact be very complicated. Although the existence of pre-nucleation clusters in highly supersaturated solution has been reported [4–6], their influence on the CaP crystal formation and whether the nucleation behavior can

be fully described by classical nucleation theory in the Ca-P system are still currently under debate and is beyond the scope of this paper [4,7,8]. Nevertheless, one should be aware of the complicated energy landscape and the presence of various possible nucleation species in the Ca-P system. With the introduction of external surfaces, different crystalline phases could precipitate and are not limited to OCP and HA. The question arises here that if multiple events that result in a sudden immobilization of free calcium ions are observed, which event should be used to signify bioactivity? In this work, we use the first major drop in free Ca^{2+} as our characteristic event to calculate supersaturation. In fact, this is an underestimation for the activities of Ti NaOH and Ti NaOH HT as this peak in the free calcium profile actually indicates the final transformation to HA. Practically, one can also use the last peak in the free calcium profile, which signifies the transformation to the thermodynamically most stable phase HA, as an indicator for bioactivity. The calculation of supersaturation takes into account of adsorbed Ca^{2+} and the change in pH and thus is a fair criterion to use for comparison between materials. For simplicity, one can also refer to the time point of the HA transformation during titration, the earlier the transformation, the less calcium is needed in the system to achieve this transformation, the higher the bioactivity. It is worth mentioning that it comes as no surprise that when HA itself is tested, there's no nucleation barrier and thus no peaks is observed in the free calcium profile. Instead, the concentration of complexed & precipitated Ca continues to rise as the titration continues, as shown in Supplementary Fig. 20.

It should also be noted, in addition to the interpretation of the free calcium profile, that the current method also relies on having sufficient surface area in the titration system. The use of powders generally provide enough surface area for heterogeneous nucleation and changes in the free calcium profile can be clearly observed. In the case of bulk samples with low surface area, the titration rate can be reduced to increase the sensitivity of the method.

Choice of animal models

Although animal models are able to simulate the biomechanics and physiology of humans, it should be remembered that they are still an approximation with animal-specific advantages and disadvantages. The sheep model is chosen in this study for its good simulation in terms of body weight, bone shape and dimensions to humans. The pelvic model of the sheep was well suited for this study as it was possible to test 96 implants with a small number of eight sheep. Unlike in different mandibular models, localization of the pelvic bone allows the assessment of osseointegration in healthy and unresponsive bone [9,10]. However, compared to models such as rats, the higher internal variance within species is a potential drawback. In the results section, some higher order interactions between factors are treated as noise due to possible internal variances between each individual sheep, although this does not change the conclusions on the main factors and represent only a minor detail in data presentation. Nevertheless, the results from the sheep model is a strong support to the *in vitro* protocol proposed in this work.

Surface roughness of the implant used for sheep experiment

The surface roughness of the implant can be investigated using surface profilometry. 3D surface profiles of four different titanium surfaces are given in Supplementary Fig. 16. At an area of ~ 1.0 x 1.5 mm, the curvature of the side surface of the Ti bar can be seen. Generally, line scratches perpendicular to the axis of the Ti bar can be observed on all surfaces with sizes of the features in the range of microns. This is likely related to the production technique and the rough features indicate a lack of fine polishing of the bars. On top of this rough surface, the effect of heat treatment and particularly NaOH treatment, which is known to induce the formation of fine porous surface layer, is however largely masked. Visual

inspection of the 3D profiles is only able to distinguish the feature of the native surface rather than any difference from chemical treatment. A quantitative analysis using line profiles all over these four surfaces revealed little difference in the average roughness values. R_a is about 1.5 μm while R_q is near 1.8 μm for all four surfaces. The impurities on the surface of Ti are likely responsible for the higher error of the roughness. However, the NaOH treatment seems to even lower the surface roughness compared to heat treatment. This is likely due to the rough surface that has masked the effect of NaOH treatment and the macroscopic area chosen in the case of Ti NaOH happens to be smoother than the area for Ti HT. No significant positive effects on surface roughness from NaOH treatment were observed.

Supplementary References

1. Vereecke, G. & Lemaître, J. Calculation of the solubility diagrams in the system $Ca(OH)_2$-H_3PO_4-KOH-HNO_3-CO_2-H_2O. *J. Cryst. Growth* **104,** 820–832 (1990).

2. Robinson, R. A. & Stokes, R. H. *Electrolyte Solutions: Second Revised Edition*. *Electrolyte Solutions: Second Revised Edition* (2002).

3. Montgomery, D. C. *Design and Analysis of Experiments, 5th Edition*. *America* (Wiley, 2000). doi:978-0-470-56319-9

4. Habraken, W. J. E. M. *et al.* Ion-association complexes unite classical and non-classical theories for the biomimetic nucleation of calcium phosphate. *Nat. Commun.* **4,** 1507 (2013).

5. Bertram, A. K., Koop, T., Molina, L. T. & Molina, M. J. Clustering of Calcium Phosphate in the System $CaCl_2$-H_3PO_4-KCl-H_2O. *J. Phys. Chem. B. Phys. Chem. B* **103,** 8230–8235 (1999).

6. Dey, A. *et al.* The role of prenucleation clusters in surface-induced calcium phosphate crystallization. *Nat. Mater.* **9,** 1010–1014 (2010).

7. De Yoreo, J. J. *et al.* Crystallization by particle attachment in synthetic, biogenic, and geologic environments. *Science.* **349,** aaa6760 (2015).

8. Carino, A., Ludwig, C., Cervellino, A., Müller, E. & Testino, A. Formation and transformation of calcium phosphate phases under biologically relevant conditions: Experiments and modelling. *Acta Biomater.* **74,** 478–488 (2018).

9. Stübinger, S. *et al.* Comparison of Er:YAG laser, piezoelectric, and drill osteotomy for dental implant site preparation: A biomechanical and histological analysis in sheep. *Lasers Surg. Med.* **42,** 652–661 (2010).

10. Ernst, S. *et al.* Comparison of two dental implant surface modifications on implants with same macrodesign: An experimental study in the pelvic sheep model. *Clin. Oral Implants Res.* **26,** 898–908 (2015).
