# Supplementary material for: Rapid evaluation of bioactive Ti-based surfaces using an in vitro titration method
Source: Nat Commun. 2019 May 2;10:2062. doi: 10.1038/s41467-019-09673-1 (PMC6497645; doi:10.1038/s41467-019-09673-1)
Supplement: Supplementary file 3 — Reporting Summary [file 41467_2019_9673_MOESM3_ESM.pdf]

nature research

Corresponding author(s): DBPR NCOMMS-18-27317

Last updated by author(s): Mar 5, 2019

Reporting Summary

Nature Research wishes to improve the reproducibility of the work that we publish. This form provides structure for consistency and transparency in reporting. For further information on Nature Research policies, see Authors & Referees and the Editorial Policy Checklist.

Statistics

For all statistical analyses, confirm that the following items are present in the figure legend, table legend, main text, or Methods section.

n/a	Confirmed	
☐	☒	The exact sample size (*n*) for each experimental group/condition, given as a discrete number and unit of measurement
☐	☒	A statement on whether measurements were taken from distinct samples or whether the same sample was measured repeatedly
☐	☒	The statistical test(s) used AND whether they are one- or two-sided<br>*Only common tests should be described solely by name; describe more complex techniques in the Methods section.*
☒	☐	A description of all covariates tested
☒	☐	A description of any assumptions or corrections, such as tests of normality and adjustment for multiple comparisons
☐	☒	A full description of the statistical parameters including central tendency (e.g. means) or other basic estimates (e.g. regression coefficient) AND variation (e.g. standard deviation) or associated estimates of uncertainty (e.g. confidence intervals)
☒	☐	For null hypothesis testing, the test statistic (e.g. *F*, *t*, *r*) with confidence intervals, effect sizes, degrees of freedom and *P* value noted<br>*Give P values as exact values whenever suitable.*
☒	☐	For Bayesian analysis, information on the choice of priors and Markov chain Monte Carlo settings
☐	☒	For hierarchical and complex designs, identification of the appropriate level for tests and full reporting of outcomes
☒	☐	Estimates of effect sizes (e.g. Cohen's *d*, Pearson's *r*), indicating how they were calculated

Our web collection on statistics for biologists contains articles on many of the points above.

Software and code

Policy information about availability of computer code

Data collection	Mettler Toledo SevenExcellence system software for pH/mV conductivity data collection, Torsion-Measurement-Unit (Model D-2209, Lorenz Messtechnik) for torque data collection
Data analysis	ImageJ, Microsoft Excel

For manuscripts utilizing custom algorithms or software that are central to the research but not yet described in published literature, software must be made available to editors/reviewers. We strongly encourage code deposition in a community repository (e.g. GitHub). See the Nature Research guidelines for submitting code & software for further information.

Data

Policy information about availability of data

All manuscripts must include a data availability statement. This statement should provide the following information, where applicable:

- Accession codes, unique identifiers, or web links for publicly available datasets
- A list of figures that have associated raw data
- A description of any restrictions on data availability

The data that support the findings of this study are available from the corresponding author upon reasonable request.

Field-specific reporting

Please select the one below that is the best fit for your research. If you are not sure, read the appropriate sections before making your selection.

☒ Life sciences ☐ Behavioural & social sciences ☐ Ecological, evolutionary & environmental sciences

For a reference copy of the document with all sections, see nature.com/documents/nr-reporting-summary-flat.pdf

Life sciences study design

All studies must disclose on these points even when the disclosure is negative.

Sample size	Sample sizes were chosen to have sufficient statistical power. For animal experiments, multi-factorial statistical design is used to minimize the number of animals used while keeping a sufficient statistical power. But the sample size is not predetermined
Data exclusions	All animal data are used. Some titration data were not used due to exceeding pre-specified experimental condition (e.g. temperature)
Replication	Successful replications were performed which contribute to the statistical significance of the findings
Randomization	Full randomization was used
Blinding	The investigators were not blinded due to practical limitations

Reporting for specific materials, systems and methods

We require information from authors about some types of materials, experimental systems and methods used in many studies. Here, indicate whether each material, system or method listed is relevant to your study. If you are not sure if a list item applies to your research, read the appropriate section before selecting a response.

Materials & experimental systems

n/a	Involved in the study	
☒	☐	Antibodies
☐	☒	Eukaryotic cell lines
☒	☐	Palaeontology
☐	☒	Animals and other organisms
☒	☐	Human research participants
☒	☐	Clinical data

Methods

n/a	Involved in the study	
☒	☐	ChIP-seq
☒	☐	Flow cytometry
☒	☐	MRI-based neuroimaging

Eukaryotic cell lines

Policy information about cell lines

Cell line source(s)	Primary osteoblast lineage cells are from mouse calvariae using sequential collagenase digestion. The C57Bl/6J mice were obtained from Charles River Laboratories in Sulzfeld, Germany. We ordered pregnant female mice and used the litters 1-2 days after birth.
Authentication	Cells were freshly prepared from donor animals, they usually are tested for their osteoblastic properties and for their capacity to support the development of osteoclasts in vitro.
Mycoplasma contamination	Primary cells are not tested for Mycoplasma.
Commonly misidentified lines (See ICLAC register)	No lines were used for these experiments.

Animals and other organisms

Policy information about studies involving animals; ARRIVE guidelines recommended for reporting animal research

Laboratory animals	Full information given in "Methods - Experimental animals and surgical model" section.
Wild animals	This study did not involve wild animals.
Field-collected samples	This study did not involve samples collected form the field.
Ethics oversight	Experiments were conducted according to Swiss laws of animal protection and welfare and approved by the local federal veterinary council (authorization: ZH108/16).

Note that full information on the approval of the study protocol must also be provided in the manuscript.
